# A comparison of two insulin infusion protocols in the medical intensive care unit by continuous glucose monitoring

**DOI:** 10.1186/s13613-016-0214-9

**Published:** 2016-11-22

**Authors:** Christophe E. M. De Block, Peter Rogiers, Philippe G. Jorens, Tom Schepens, Cosimo Scuffi, Luc F. Van Gaal

**Affiliations:** 1 Department of Endocrinology, Diabetology and Metabolism, Faculty of Medicine, Antwerp University Hospital and University of Antwerp, Wilrijkstraat 10, 2650 Edegem, Belgium; 2 Intensive Care Unit, ZNA, General Hospital Middelheim, Antwerp, Belgium; 3 Intensive Care Unit, Antwerp University Hospital, University of Antwerp, Edegem, Belgium; 4 A. Menarini Diagnostics, Scientific and Technology Affairs, Florence, Italy

**Keywords:** Intensive care unit, Continuous glucose monitoring, Insulin infusion protocol, Hypoglycemia

## Abstract

**Background:**

Achieving good glycemic control in intensive care units (ICU) requires a safe and efficient insulin infusion protocol (IIP). We aimed to compare the clinical performance of two IIPs (Leuven versus modified Yale protocol) in patients admitted to medical ICU, by using continuous glucose monitoring (CGM). This is a pooled data analysis of two published prospective randomized controlled trials. CGM monitoring was performed in 57 MICU patients (age 64 ± 12 years, APACHE-II score 28 ± 7, non-diabetic/diabetic: 36/21). The main outcome measures were percentage of time in normoglycemia (80–110 mg/dl) and in hypoglycemia (<60 mg/dl), and glycemic variability (standard deviation, coefficient of variation, mean amplitude of glucose excursions, mean of daily differences).

**Results:**

Twenty-two subjects were treated using the Leuven protocol and 35 by the Yale protocol; >63,000 CGM measurements were available. The percentage of time in normoglycemia (80–110 mg/dl) was higher (37 ± 15 vs. 26 ± 11%, *p* = 0.001) and percentage of time spent in hypoglycemia was lower (0[0–2] vs. 5[1–8]%, *p* = 0.001) in the Yale group. Median glycemia did not differ between groups (118[108–128] vs. 128[106–154] mg/dl). Glycemic variability was less pronounced in the Yale group (median SD 28[21–37] vs. 47[31–71] mg/dl, *p* = 0.001; CV 23[19–31] vs. 36[26–50]%, *p* = 0.001; MODD 35[26–41] vs. 60[33–94] mg/dl, *p* = 0.001). However, logistic regression could not identify type of IIP, diabetes status, age, BMI, or APACHE-II score as independent parameters for strict glucose control.

**Conclusions:**

The Yale protocol provided better average glycemia, more time spent in normoglycemia, less time in hypoglycemia, and less glycemic variability than the Leuven protocol, but was not independently associated with strict glycemic control.

**Electronic supplementary material:**

The online version of this article (doi:10.1186/s13613-016-0214-9) contains supplementary material, which is available to authorized users.

## Background

Consensus exists that overt hyperglycemia (>150 mg/dl) in patients admitted to the intensive care unit (ICU) should be treated to improve morbidity and survival [1]. However, there is little agreement on the ideal target range of glycemia [2]. Strict glycemic control (80–110 mg/dl) is no longer recommended for most ICUs, but in highly standardized ICUs a strict target may be feasible without increasing hypoglycemia. Achieving strict glycemic control is a complex task since during ICU stay severity of illness and degree of insulin resistance may fluctuate, nutritional delivery may change, and interventions (e.g., administration of corticosteroids) may produce frequent changes in insulin needs [2]. Therefore, multiple insulin infusion protocols (IIPs) were created, all meant to balance efficacy with safety (avoid hypoglycemia), and attainability (nursing workload).

So far, no single IIP has been established as the most effective for obtaining tight glycemic control. Earlier observational studies and randomized controlled trials (RCTs) in medical ICU (MICU) or mixed ICU settings, and targeting a glycemia between 80 and 110 mg/dl, reported that 22–60% of all blood glucose values were in target for paper-based IIPs [3–14], compared to 42–69% for computerized decision-supported algorithms [4, 10, 11, 14, 15]. The efficacy and safety of different IIPs on glycemic control have recently been investigated using computer simulation models [16, 17] and in RCTs in cardiac surgery patients [18, 19]. However, to the best of our knowledge, comparing the effect of different IIPs on glucose control has never been investigated in MICU patients. With this pooled data analysis of two published prospective RCTs [5, 6], we assessed the clinical performance of two IIPs (Leuven versus modified Yale protocol) in patients admitted to MICU, by means of continuous glucose monitoring (CGM), allowing a complete picture of glucometrics.

## Methods

This is a pooled data analysis of two prospective RCTs conducted at the medical ICUs of the Antwerp University Hospital (45 beds, including 14 MICU beds) and Middelheim Hospital, a university-affiliated tertiary care center (36 beds, including 13 MICU beds) [5, 6]. Patients were recruited between 04/2004 and 03/2005 for the first study and between 07/2007 and 09/2009 for the second study. Both MICUs applied the same standards of care, with a nurse-to-patient ratio between 1:2.5 and 1:3.0. Each patient or the closest family member gave written informed consent. Patients were included if they were between 18 and 75 years, treated by IV insulin, and expected to stay in MICU for ≥3 days. Patients were not enrolled if pregnant, if surgery was the reason for admission, or if a do not reanimate code was present. The studies were approved by the ethics committees of both hospitals (Middelheim approval no. 2345 and UZA 6/43/211) and conducted in accordance with the amended Declaration of Helsinki.

Severity of illness was scored using the Acute Physiology And Chronic Health Evaluation II (APACHE-II) and the Sequential Organ Failure Assessment (SOFA) score [20, 21]. The neurologic score was zero when patients were sedated. Enteral nutrition was started as soon as possible, at 25 kcal/kg body weight per day.

### Glucose monitoring

Forty-eight-hour CGM was initiated within the first 48 h after admission using a microdialysis-based device that is not equipped with alarms (GlucoDay^®^ in the first study and GlucoDay^®^S in the second study, A. Menarini Diagnostics, Florence, Italy). The methodology has been described before [5, 6]. Briefly, a microdialysis fiber (Medica, Medolla, Italy) was inserted subcutaneously into the periumbilical region using an 18-gauge Teflon^®^ (DuPont, Wilmington, DE) catheter as a guide. The device does not use any coagulant. The device uses a glucose oxidase-based amperometric biosensor to measure glucose concentrations in the interstitial dialysate every 3 min over a 48 h period.

Data analysis for accuracy and glucometrics was performed by applying, in silico to the CGM signal, a two-point calibration according to the manufacturer’s requirements (one calibration every 24 h). This was performed in order to avoid an overestimation of the system’s accuracy if one uses a higher number of calibration points. In the RT-CGM group, however, to be as safe as possible and to account for possible changes in subcutaneous glucose recovery due to hemodynamic alterations (e.g., hypotension, shock, vasoactive drugs), for each sensor, a six-point calibration was performed (after 2, 6, 12, 18, 24, and 36 h) using arterial BG values, but these data were not used for statistical analysis [6]. This was done to assure that the glucose readings and trends shown by RT-CGM would be clinically reliable. At present, RT-CGM is becoming more common practice, but at the time of the study, a real-time CGM device was not approved to make clinical adjustments of insulin therapy, and our ethical committee would not approve clinical decisions to be made solely on the basis of RT-CGM at that time. In order to avoid clinical decisions being made on potentially inaccurate CGM data, nurses had to take an additional arterial blood glucose sample. Thus, direct corroboration of the data was explicitly needed. The RT-CGM system was thus used only as a prompt to take an extra blood glucose sample if the rate of change in glucose exceeded 25 mg/dl per 30 min. Since no differences in glucometrics were observed in the REGIMEN trial between in patients randomized to RT-CGM versus blinded CGM, we pooled the data. For all patients, adjustments of insulin therapy were made on the basis of arterial blood glucose values [6].

### Insulin infusion protocols

Twenty-two patients received continuous IV insulin (regular insulin Actrapid; Novo Nordisk, Bagsvaerd, Denmark) according to the Leuven protocol, targeting a blood glucose between 80 and 110 mg/dl [5, 13]. Thirty-five subjects were treated with IV insulin (insulin aspart, Novo Nordisk, Bagsvaerd, Denmark) according to a modified Yale protocol, targeting a blood glucose level between 80 and 120 mg/dl [6].

In both groups, arterial blood glucose levels were measured using an on-site blood gas analyzer (Rapidlab^®^ 1265, Siemens, München, Germany) and they were used to adjust the insulin infusion rate. Insulin in a concentration of 50 units in 50 cc 0.9% NaCl was infused using the Injectomat Agilia^®^ syringe infusion system (flow rate change: range 0.1–200 ml/h, Fresenius Kabi, Bad Homburg, Germany). In both groups, the arterial blood glucose sampling interval varied between 1 and 4 h. For more details on the insulin infusion protocols, the reader is referred to the original publications [5, 6].

### Outcome parameters

The percentage of time spent in the target range of glycemia (80–110 mg/dl) was the primary outcome parameter. Secondary outcome variables were percentages of time spent in hypoglycemia (<60 mg/dl), in hyperglycemia (>150 and >200 mg/dl), and parameters of glycemic fluctuations (SD: standard deviation [22], CV: coefficient of variation [22], MAGE: mean amplitude of glucose excursions [23], CONGA: continuous overlapping net glycemic action [24], and MODD: mean of daily differences [25]. We also calculated the low blood glucose index (LBGI) and the high blood glucose index (HBGI) as measures of risk of hypo- and hyperglycemia [26]. All glucometric data reported are those calculated using CGM data. A CGM reading <60 mg/dl, confirmed by an arterial blood glucose sample <60 mg/dl, lasting for >6 min (6 min being the time of two CGM measurements) was defined as a hypoglycemic event. We rigorously followed the recommendations on measurement of blood glucose and reporting glycemic control in critically ill adults [27].

### Statistical analysis

Results were analyzed using SPSS (SPSS Inc., Chicago, USA). Distributions of continuous data were tested for normality by Kolmogorov–Smirnov test. The unpaired *t* test or Mann–Whitney *U* test were used to determine differences between groups, with Bonferroni adjustments for multiple comparisons. Data are expressed as mean ± SD or median [25th–75th percentile]. Differences in distributions of categorical data were evaluated by *χ* ^2^ or Fisher’s exact test. Stepwise forward logistic or linear regression analysis was performed to assess the strength and independency of associations. A two-tailed *p* < 0.05 was considered significant.

## Results

Over 63,000 CGM data points were analyzed from 57 adults (men/women: 30/27, non-diabetic/diabetic: 36/21) admitted to the MICU. Mean age was 64 ± 12 years. They were severely ill as demonstrated by a mean APACHE-II score of 28 ± 7 and SOFA score of 10 ± 4. Table 1 shows reasons for admission and interventions used.

**Table 1:** Baseline characteristics, interventions used and glucometrics of patients treated by the Leuven versus Yale protocol

	Total cohort	Leuven	Yale	Statistics
Number of patients	57	22	35	*p* value
Patient demographics				
Men/women	30/27	13/9	17/18	NS
Diabetic status (no/type1/type2)	36/6/15	9/4/9	27/2/6	0.0016
Age (years)	64 ± 12	60 ± 13	66 ± 10	0.055
BMI (kg/m^2^)	26.8 ± 5.4	27.5 ± 7.1	26.3 ± 4.0	NS
Admission reason				
Septic shock	22	13	9	NS
Neurologic disease/coma	9	0	9	0.004
Cardiopulmonary resuscitation	9	1	8	NS
Respiratory failure	6	1	5	NS
Cardiogenic shock	7	3	4	NS
Other	4	4	0	NS
Severity of illness				
APACHE-II score	28 ± 7	27 ± 7	28 ± 7	NS
SOFA score	10 ± 4	9 ± 4	11 ± 3	0.072
Clinical interventions				
Mechanical ventilation	44	16	28	NS
Vasopressor therapy	35	14	21	NS
Inotropic therapy	20	6	14	NS
Hemodialysis	14	9	5	NS
No/total parenteral/enteral feeding	17/17/23	9/10/3	8/7/20	0.003
Glucocorticoids	27	12	15	NS
Blood transfusion	15	4	11	NS
Antibiotics	46	19	27	NS
Outcome parameters				
LOS in ICU (days)	15 ± 9	11 ± 6	17 ± 10	0.019
In hospital mortality	20	7	11	NS
Insulin dose				
Day 1 (units)		138 (48–190)	50 (27–80)	0.001
Day 2 (units)		116 (54–116)	56 (28–81)	0.006
Glucose parameters				
HbA1c (%)		6.3 (5.8–7.0)	6.0 (5.7–6.6)	NS
HbA1c (mmol/mol)		45 (40–53)	42 (39–49)	NS
Median glycemia (mg/dl)		128 (106–154)	118 (108–128)	NS
% of time at glycemia				
<60 mg/dl		5 (1–8)	0 (0–2)	0.001
80–110 mg/dl		26 ± 11	37 ± 15	0.001
>150 mg/dl		29 ± 23	17 ± 13	<0.0001
>200 mg/dl		13 ± 19	3 ± 5	<0.0001
60–150 mg/dl		66 ± 24	81 ± 7	<0.0001
70–180 mg/dl		69 ± 19	91 ± 10	<0.0001
Nr of art blood glc measurements/day		10 ± 2	10 ± 4	NS
Glucose variability parameters				
SD (mg/dl)		47 (31–71)	28 (21–37)	0.001
Coefficient of variation (%)		36 (26–50)	23 (19–31)	0.001
IQR		66 (47–82)	37 (27–43)	<0.0001
MAGE (mg/dl)		73 (47–128)	52 (37–83)	0.061 (NS)
MODD (mg/dl)		60 (33–94)	35 (26–41)	0.001
M-100		10 (4–25)	4 (2–5)	<0.0001
CONGA1 (mg/dl)		20 (14–39)	15 (13–24)	NS
CONGA2 (mg/dl)		30 (22–53)	23 (18–34)	0.036
CONGA4 (mg/dl)		38 (28–66)	29 (20–43)	0.012
LBGI		2.6 (1.0–3.7)	0.7 (0.4–1.4)	0.011
HBGI		2.5 (1.0–8.0)	1.3 (0.5–1.8)	0.018
Glucose variability		42 (29–67)	28 (20–34)	0.001

Data are presented as numbers, as mean ± SD or median (25–75th percentile)

*LOS in ICU* length of stay in ICU, *IQR* interquartile range, *MAGE* mean amplitude of glycemic excursions, *MODD* mean of daily differences, *LBGI* low blood glucose index, *HBGI* high BGI

### Comparison of Leuven versus Yale protocol

Twenty-two subjects were treated using the Leuven protocol and 35 by the Yale protocol. The distribution of diabetic subjects differed between groups (13/22 Leuven vs. 8/35 Yale; *p* = 0.0016). Patients in the Yale group tended to be older. Reasons for admission were comparable between groups with exception of neurologic disease/coma. Feeding habits were different in the two groups (*p* = 0.003) with enteral feeding being less frequently used in the Leuven group (3/22 vs. 20/35). The APACHE-II score was similar, whereas the SOFA score tended to be higher in the Yale group.

**Fig. 1:**
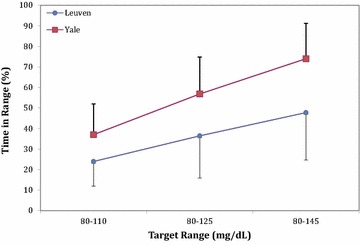
Average percentage time in range and SD over the groups (Yale vs. Leuven) for the different glycemia target ranges. *P* < 0.0001 for all three ranges

**Fig. 2:**
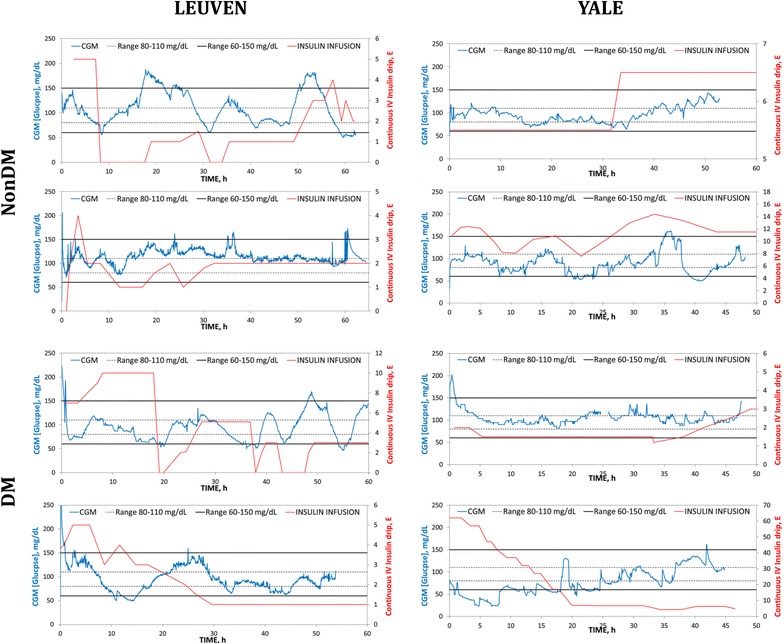
Insulin glucose plots comparing 4 non-diabetic and 4 diabetic patients treated according to the Leuven protocol versus according to the modified Yale protocol. *CGM* continuous glucose monitoring

Patients in the Leuven group required much more IV insulin compared to those treated by the Yale protocol (Table 1). Despite a similar number of arterial blood glucose measurements, patients in the Yale protocol had better glucometrics with a higher percentage of time in target glycemia (80–110 mg/dl) (37 ± 15 vs. 26 ± 11%, *p* = 0.001) and a lower percentage of time spent in hypoglycemia (0[0–2] vs. 5[1–8]%, *p* = 0.001). Also percentage of time spent between 60 and 150 mg/dl (81 ± 7 vs. 66 ± 24%, *p* < 0.0001), 80–125 mg/dl (57 ± 18 vs. 36 ± 21%, *p* < 0.0001), between 80 and 145 mg/dl (74 ± 17 vs. 48 ± 23, *p* < 0.0001), and between 70 and 180 mg/dl (91 ± 10 vs. 69 ± 19%, *p* < 0.0001) was higher in the Yale group. Figure 1 shows the time-in-band for the different ranges of targets for both groups. Median glycemia, however, did not differ between groups (118[108–128] (log: 114[105–125]) vs. 128[106–154] (log: 122[99–136]) mg/dl) (Table 1). Glycemic variability was less pronounced with the use of the Yale protocol (median[IQR] SD 28[21–37] vs. 47[31–71] mg/dl, *p* = 0.001; median CV 23[19–31] vs. 36[26–50]%, *p* = 0.001; median MODD 35[26–41] vs. 60[33–94] mg/dl, *p* = 0.001). Significant better LBGI and HBGI were observed in the Yale group (Table 1). Eight insulin/glucose plots comparing non-diabetic and diabetic patients treated according to the Leuven protocol versus according to the modified Yale protocol are shown in Fig. 2, providing the reader with a good visual image.

### Characteristics of subjects achieving strict versus above-target glycemic control

Subjects achieving strict glycemic control (*n* = 19), defined as having an average glycemia ≤110 mg/dl, did not differ with regard to gender, diabetic status, age, BMI, reason for admission, severity of illness, and interventions used as compared to the ones not obtaining an average glycemia ≤110 mg/dl, with the exception of the use of glucocorticoids (*p* = 0.001) (Table 2). The distribution according to insulin infusion protocol used (Leuven vs. Yale) also did not differ between groups. Insulin doses infused and a number of arterial blood glucose measurements were similar as well. Most glucometrics (% of time within target) including glycemic variability (SD, MODD, LBGI and HBGI) were better in the group achieving strict glycemic control. Logistic regression analysis could not identify type of IIP, diabetes status, age, BMI, or APACHE-II score as independent parameters for strict glucose control. The only parameter which showed an independent association with strict glucose control was the administration of glucocorticoids (*p* = 0.001).

**Table 2:** Baseline characteristics, interventions used and glucometrics of patients reaching an average glycemia ≤110 mg/dl versus those with an average glycemia >110 mg/dl

	Avg glyc ≤ 110 mg/dl	Avg glyc >110 mg/dl	Statistics
Number of patients	19	38	
Patient demographics			
Men/women	8/11	22/16	NS
Diabetic status (no/type1/type2)	14/1/4	23/4/11	NS
Age (years)	65 ± 11	63 ± 12	NS
BMI (kg/m^2^)	26.3 ± 3.8	27.1 ± 6.1	NS
Severity of illness			
APACHE-II score	27 ± 6	28 ± 7	NS
SOFA score	10 ± 4	10 ± 3	NS
Clinical interventions			
Mechanical ventilation	14	31	NS
Vasopressor therapy	12	23	NS
Hemodialysis	5	9	NS
No/total parenteral/enteral feeding	4/6/9	13/11/14	NS
Glucocorticoids	15	12	0.01
Antibiotics	17	29	NS
Protocol (Leuven/Yale)	7/12	15/23	NS
Insulin dose			
Day 1 (units)	62 (33–133)	63 (39–128)	NS
Day 2 (units)	67 (23–101)	70 (42–120)	NS
Glucose parameters			
HbA1c (%)	6.1 (5.6–6.7)	6.0 (5.8–6.9)	NS
HbA1c (mmol/mol)	43 (38–50)	70 (42–120)	NS
Median glycemia (mg/dl)	104 (100–108)	128 (119–141)	<0.0001
% of time at glycemia			
<60 mg/dl	5 (0–8)	0 (0–4)	0.067
80–110 mg/dl	41 ± 14	27 ± 13	0.001
>150 mg/dl	6 ± 6	28 ± 18	<0.0001
>200 mg/dl	1 ± 2	10 ± 16	0.017
60–150 mg/dl	89 ± 11	69 ± 19	<0.0001
70–180 mg/dl	86 ± 14	80 ± 19	NS
Nr of art blood glc measurements/day	10 ± 3	10 ± 4	NS
Glucose variability parameters			
SD (mg/dl)	25 (20–36)	38 (29–63)	0.003
Coefficient of variation (%)	25 (20–33)	29 (23–44)	NS
IQR	33 (27–55)	45 (35–76)	0.031
MAGE (mg/dl)	50 (34–72)	60 (43–100)	0.085
MODD (mg/dl)	29 (22–45)	42 (32–76)	0.005
M-100	2 (1–4)	6 (4–14)	0.001
CONGA1 (mg/dl)	15 (12–19)	21 (15–35)	0.008
CONGA2 (mg/dl)	22 (17–28)	32 (20–48)	0.010
CONGA4 (mg/dl)	29 (18–43)	42 (27–69)	0.011
LBGI	2.7 (0.9–3.8)	0.7 (0.2–1.9)	0.001
HBGI	0.3 (0.2–1.1)	2.0 (1.4–4.5)	<0.0001
Glucose variability	21 (17–28)	36 (29–53)	<0.0001

Data are presented as numbers, as mean ± SD or median (25–75th percentile)

*LOS in ICU* length of stay in ICU, *IQR* interquartile range, *MAGE* mean amplitude of glycemic excursions, *MODD* mean of daily differences, *LBGI* low blood glucose index, *HBGI* high BGI

### Characteristics of diabetic versus non-diabetic subjects

No differences in patient demographics except for BMI (*p* = 0.02), in reason for admission, severity of illness, and clinical interventions used, were present between diabetic (*n* = 21) and non-diabetic critically ill patients (Table 3). There were more diabetic patients in the Leuven protocol (*p* = 0.009). Diabetic subjects required more insulin, had a worse median glycemia (131[110–166] vs. 116[107–128] mg/dl, *p* = 0.034), spent less time in target glycemia (25 ± 12 vs. 36 ± 15%, *p* = 0.006), more time in hypoglycemia (4[1–10] vs. 0[0–1]%, *p* = 0.001) than non-diabetic subjects. All other glucometrics, including glycemic variability parameters, were worse as well in diabetic patients (see Table 3).

**Table 3:** Baseline characteristics, interventions used and glucometrics of non-diabetic versus diabetic patients

	Non-DM	DM	Statistics
Number of patients	36	21	
Patient demographics			
Men/women	19/17	11/10	NS
Age (years)	65 ± 11	62 ± 12	NS
BMI (kg/m^2^)	26 ± 4	29 ± 7	0.02
Severity of illness			
APACHE-II score	29 ± 6	26 ± 7	NS
SOFA score	11 ± 4	9 ± 3	NS (0.064)
Clinical interventions			
Mechanical ventilation	27	17	NS
Vasopressor therapy	23	12	NS
Hemodialysis	12	2	NS (0.06)
No/total parenteral/enteral feeding	7/13/16	10/4/7	NS (0.075)
Glucocorticoids	20	7	NS
Antibiotics	30	16	NS
Protocol (Leuven/Yale)	9/27	13/8	0.009
Insulin dose			
Day 1 (units)	48 (26–82)	127 (51–175)	0.001
Day 2 (units)	46 (25–85)	113 (67–168)	0.009
Glucose parameters			
HbA1c (%)	5.9 (5.6–6.3)	6.9 (6.1–7.3)	<0.0001
HbA1c (mmol/mol)	41 (38–45)	52 (43–56)	<0.0001
Median glycemia (mg/dl)	116 (107–128)	131 (110–166)	0.034
% of time at glycemia			
<60 mg/dl	0 (0–1)	4 (1–10)	0.001
80–110 mg/dl	36 ± 15	25 ± 12	0.006
>150 mg/dl	32 ± 15	24 ± 11	0.001
>200 mg/dl	3 ± 15	15 ± 20	0.001
60–150 mg/dl	83 ± 12	63 ± 23	<0.0001
70–180 mg/dl	90 ± 11	69 ± 19	<0.0001
Nr of art blood glc measurements/day	10 ± 4	10 ± 3	NS
Glucose variability parameters			
SD (mg/dl)	28 (21–35)	53 (40–75)	<0.0001
Coefficient of variation (%)	23 (19–30)	38 (29–51)	<0.0001
IQR	38 (28–45)	66 (41–108)	<0.0001
MAGE (mg/dl)	49 (35–66)	87 (56–127)	0.002
MODD (mg/dl)	35 (26–42)	59 (35–116)	<0.0001
M-100	4 (2–5)	12 (6–29)	<0.0001
CONGA1 (mg/dl)	15 (13–20)	30 (19–41)	<0.0001
CONGA2 (mg/dl)	22 (17–30)	44 (26–59)	<0.0001
CONGA4 (mg/dl)	30 (21–43)	60 (37–86)	<0.0001
LBGI	0.7 (0.3–1.8)	2.3 (0.9–3.7)	0.013
HBGI	1.2 (0.4–1.8)	3.0 (1.4–10.3)	0.002
Glucose variability	28 (20–34)	44 (34–79)	<0.0001

Data are presented as numbers, as mean ± SD or median (25–75th percentile)

*LOS in ICU* length of stay in ICU, *IQR* interquartile range, *MAGE* mean amplitude of glycemic excursions, *MODD* mean of daily differences, *LBGI* low blood glucose index, *HBGI* high BGI

In the non-diabetic group, patients in the Yale protocol spent more time in target glycemia (40 ± 15 vs. 25 ± 10%, *p* = 0.009), less time in hypoglycemia (0[0–0] vs. 1[0–6]%, *p* = 0.013), and glycemic variability tended to be smaller (SD *p* = 0.076, CV *p* = 0.057, MODD *p* = 0.021) than those treated by the Leuven protocol. Median glycemia was similar (117[108–127] vs. 115[101–140]) in both groups (Additional file 1: Table S1). In the diabetic subjects, insulin needs were lower (*p* = 0.044) and patients spent less time at a glycemia >150 mg/dl (26 ± 21 vs. 35 ± 25%, *p* = 0.003) in the Yale compared to the Leuven group. However, median glycemia (120[108–140] vs. 136[111–169] mg/dl), time spent in hypoglycemia or at target range, and parameters of glycemic variability were similar between groups (Additional file 1: Table S1).

## Discussion

Achieving strict glycemic control without risk of hypoglycemia in the ICU is difficult. It requires a comprehensive and safe insulin infusion protocol (IIP) that is both detailed enough and practical enough to be easily implemented by ICU nurses [2, 28]. Multiple IIPs have been developed, but to the best of our knowledge this is the first study comparing the clinical efficacy (% time in target glycemia) and safety (hypoglycemia, glycemic variability) of two IIPs in MICU patients by means of CGM. Overall, compared to existing data (see Table 4, [3–15, 29–38], both our IIPs were able to obtain reasonably strict glucose control without excessive risk of hypoglycemia. The percentage of time in normoglycemia was higher (37 vs. 26%), and percentage of time in hypoglycemia lower (0 vs. 5%) and glycemic variability was less pronounced in patients treated with the Yale IIP. Diabetes status can, however, be a confounding factor [34]. We observed an imbalance in the number of diabetic subjects with more diabetic patients in the Leuven group. Diabetic as compared to non-diabetic subjects required more insulin, had worse glycemic control, and larger glycemic variability, thereby possibly blunting the effect of the IIP. However, logistic regression could not identify type of IIP, diabetes status, or severity of illness as independent parameters associated with strict glucose control. When comparing Yale versus Leuven protocol in non-diabetic subjects, patients in the Yale protocol had better glucometrics. This was evident, despite the low number of patients. In the diabetic subgroup, however, the advantages of the Yale protocol were less pronounced.

**Table 4:** Summary of several insulin infusion protocols in different ICU settings

Authors	ICU type	Study	*n*	% Diabetic patients	Protocol	Target glycemia (mg/dl)	Method of glucose measurements
Goldberg et al. (2004)	MICU	Observational	52	56	Yale	100–139	Hospital glucose meter: near hourly measurements
Van den Berghe et al. (2006)	MICU	RCT	1200	17	Paper: Leuven	80–110	ABG: q1-4 h
Kulnik et al. (2008)	MICU	Observational	10	20	eMPC (computer)	80–110	Variable sampling rate: q20 min-4 h
Shetty et al. (2012)	MICU	Observational	90	66	Yale	120–160	POC meter: hourly measurements
Holzinger et al. (2010)	MICU	RCT	124	19	Leuven	80–110	CGM
De Block et al. (2015)	MICU	RCT	35	23	Yale	80–110	CGM
Finney et al. (2003)	Mixed	Observational	523	16	Paper	90–145	ABG
Juneja et al. (2007)	Mixed	Observational	2398	NR	Clarian Gluco Stabilizer	80–110	POC: q1-2 h
Chase et al. (2008)	Mixed	Observational	371	17	SPRINT	80–110	Sampling rate: q1-2 h
Morris et al. (2008)	Mixed	Before–after	755	NR	eProtocol-insulin versus paper	80–110	POC: q1-4 h
Preiser et al. GLUCONTROL (2009)	Mixed	RCT	1078	21	Paper: glucontrol	80–110	POC: q1-4 h
NICE SUGAR (2009)	Mixed	RCT	6104	20	Paper: Leuven	81–108	ABG
Marvin et al. (2013)	Mixed	Retrospective	1657	NR	Computerized Yale	100–140	POC: variable time interval
Van Herpe et al. (2013)	Mixed	RCT	300	21	LOGIC-insulin computerized	80–110	ABG: variable time interval: q1-4 h
Krinsley et al. (2015)	Mixed	Retrospective	3297	23	Paper: Stamford	70–140	POC: q3 h
Vogelzang et al. (2005)	SICU	Observational	179	15	GRIP	72–135	POC blood gas analyzer: variable
Plank et al. (2006)	SICU: cardiothoracic surgery	RCT	60	23	eMPC versus paper	80–110	POC: variable sampling rate: q1 h-4 h
Hovorka et al. (2007)	SICU: cardiac surgery	RCT	60	45	eMPC	80–110	Variable sampling rate: q1 h-4 h
Saager et al. (2008)	SICU: cardiothoracic ICU	RCT	40	100	EndoTool (computer) versus paper	90–150	POC: hourly
Dortch et al. (2008)	SICU: trauma ICU	RCT	552		Computer versus paper	80–110	POC q1-4 h
Blaha et al. (2009)	SICU: cardiac surgery	RCT	120	14	eMPC versus paper (Matias versus Bath)	80–110	ABG: protocol dependent: q1-4 h
Barletta et al. (2011)	SICU	Before–after	192	28	Computer versus paper	80–110	POC: variable sampling rate: q30 min-2 h versus q2 h
Dumont et al. (2012)	SICU: cardiovascular ICU	RCT	300	43	Computer (EndoTool) versus paper (modified Portland)	80–150	NA

Authors	Duration of glucose monitoring	Glucometric to measure target	% of time at target glycemia: intervention versus control group	Mean glycemia (mg/dl): intervention versus control group	Hypoglycemia: intervention versus control group	Glycemic variability	References
Goldberg et al. (2004)	61 h	Percent of hourly BG values in target range	52%	124 ± 15	% of data at glc <60 mg/dl: 0.3%	NA	[8]
Van den Berghe et al. (2006)	NR	Mean morning BG	NA	111 ± 29 versus 153 ± 31	% of patients: glc <40 mg/dl: 18.7 versus 3.1%	NA	[13]
Kulnik et al. (2008)	72 h	Percent of BG values in target	47 ± 13%	109 ± 13	% data at glc <40 mg/dl: 0%	NA	[15]
Shetty et al. (2012)	59 h	Percent of BG values in target range	42%	156 ± 23	% of data <70 mg/dl: 0.3%	NA	[37]
Holzinger et al. (2010)	72 h	CGM data: percent of data in target range	59 ± 20 versus 55 ± 18	106 ± 18 versus 111 ± 10	Rate: 1.9% versus 11.5%	NA	[9]
De Block et al. (2015)	96 h	CGM data: percent of data in target range	37 ± 12 versus 34 ± 10	119 ± 17 versus 122 ± 11	% of time at glc <60 mg/dl: 0.6 ± 1.6 versus 2.4 ± 4.3%	No differences between groups in SD, MAGE, MODD, CV	[6]
Finney et al. (2003)	22–89 h	Time spent in glucose band 80–110 mg/dl	4 (0–20)%	NR	0 ± 1%	NA	[31]
Juneja et al. (2007)	NR	Percent of data in target range	52 versus 32%	107 ± 39	% data at glc <50 mg/dl: 0.4 versus 0.5%	NA	[10]
Chase et al. (2008)	53 h	Percent of BG values in target	54%	108 ± 27	% of data at glc <72 mg/dl: 3.8%	SD: 27 mg/dl	[4]
Morris et al. (2008)	4–22 days	Percent of BG values in target	42 versus 28%	116 versus 134	% data at glc <40 mg/dl: 11.1 versus 5.1%	NA	[11]
Preiser et al. GLUCONTROL (2009)	48–216 h (=2–9 days)	Proportion of time of BG values in range	43%	117 (IQR: 108–130) mg/dl	Proportion of time at glc <40 mg/dl: 5.9 ± 27%	SD: 36 mg/dl	[12]
NICE SUGAR (2009)	4.2 days (1.9–9.0 days)	Time-weighted mean BG	NR	115 ± 18 versus 144 ± 23	% of patients: glc <40 mg/dl: 6.8 versus 0.5%	NA	[7]
Marvin et al. (2013)	NR	Percent of hourly BG values in target range	92%	124	% of data 40–70 mg/dl: 1.1% and in 17.6% of patients	NA	[34]
Van Herpe et al. (2013)	26–113 h	Percent of BG values in target range	69 ± 17 versus 60 ± 19	106 ± 9 versus 107 ± 11	% data at glc <60 mg/dl: 0.6 versus 1.2%	Max change in glc/24 h: 31 versus 37 mg/dl	[14]
Krinsley et al. (2015)	36–120 h	Percent of time of BG values in target range	Non-DM versus DM: 81 (61–94) versus 55 (35–71)%	Non-DM versus DM: 121 (112–133) versus 140 (128–155) mg/dl	% of patients: glc <70 mg/dl: non-DM versus DM: 18 versus 31%	CV: non-DM versus DM: 18 versus 27%	[33]
Vogelzang et al. (2005)	1.6 (0.8–4.7) days	Percent of time of BG values in target	78 (66–88)%	121 (108–135)	% of patients: glc <40: 0.6%; glc <63: 11.2%	NA	[38]
Plank et al. (2006)	48 h	Percent of time in target range	52 (17–92) versus 19 (0–71)%	117 (102–144) versus 131 (97–237)	Number of hypo episodes (<54 mg/dl) over 48 h: 0 versus 2	NA	[35]
Hovorka et al. (2007)	24 h	Percent of time in target range	60 ± 23 versus 28 ± 16	112 ± 20 versus 130 ± 20	% of data at glc <52 mg/dl: 0% versus 0%	NA	[32]
Saager et al. (2008)	9 h	Percent of BG values in target	84 versus 60%	126 ± 18 versus 147 ± 27	Episodes of hypo (<60 mg/dl) during ICU: 4 versus 1	NA	[36]
Dortch et al. (2008)	NR	Percent of BG values in target	42 versus 34%	116 ± 37 versus 120 ± 37	% data at glc <40 mg/dl: 0.2 versus 0.5%	NA	[30]
Blaha et al. (2009)	45–48 h	Time in target range	46 ± 3 versus 38 ± 3 versus 40 ± 3%	106 ± 4 versus 121 ± 4 versus 117 ± 4	Time in hypo (<52 mg/dl): 0 ± 0 versus 0.4 ± 0.2 versus 0.4 ± 0.3%	NA	[18]
Barletta et al. (2011)	67 versus 98 h	Percent of BG values in target	49 ± 14 versus 40 ± 12	113 ± 11 versus 116 ± 11	% data at glc <40 mg/dl: 2.1 versus 4.1%	SD: 25 ± 9 versus 31 ± 11 mg/dl	[29]
Dumont et al. (2012)	NA	Percent of BG values in target range	70 ± 15 versus 62 ± 18	138 ± 16 versus 141 ± 20	Number of hypo events <60 mg/dl: 7 (5%) versus 18 (11%)	SD:36 ± 18 versus 42 ± 21	[19]

*MICU* medical intensive care unit, *SICU* surgical ICU, *RCT* randomized controlled trial, *NR* not reported, *NA* not assessed, *ABG* arterial blood glucose, *POC* point of care, *SD* standard deviation

Up till now no single IIP has been established as the most effective for obtaining tight glycemic control [28, 39, 40]. The IIP should be tailored to the subset of patients being treated and to local resources, because an excellent validated IIP is no guarantee for optimal glucose control unless it is carefully implemented. Most IIPs show significant similarities, but differences relate to target glucose levels (80–110 mg/dl versus ranges varying between 90 and 180 mg/dl), initial glycemic threshold (>150–200 mg/dl), infusion rates, use of boluses, and frequency of monitoring. Changes in insulin infusion rate may relate to actual glycemia, direction and/or velocity of change in glycemia, degree of insulin resistance, and insulin dose. The population treated (surgical vs. medical ICU, diabetes status) may also affect the performance of the IIP [2, 28, 39, 40]. The competence of the nurses and clarity of instructions also influence outcome. Computerized decision-supported algorithms might provide superior glucose control compared to paper-based IIPs because of reduced errors by enabling the use of complex mathematical calculations and better protocol consistency. In MICUs and mixed ICUs where a glycemia between 80 and 110 mg/dl was targeted, 22–60% of all blood glucose values were reported to be in target for paper-based IIPs [3–14], compared to a higher percentage (42–69%) for computerized IIPs [4, 10, 11, 14, 15] (Additional file 1: Digital Content–Table S1). In contrast, in a before–after study in 192 surgical ICU patients, Barletta et al. [29] could not observe significant glucometric differences between the computer-assisted versus paper-based IIP. It is probably not the paper or computer that makes the largest difference; but the IIP algorithm itself and the competence of the staff.

A head-to-head comparison of different IIPs on glycemic control has only been performed in RCTs in cardiac surgery. Blaha et al. [18] compared two paper protocols with a computerized IIP in 120 patients, showing that the computerized IIP provided better average glucose control, but with a longer time in hypoglycemia risk range than the paper protocols. Dumont and Bourguignon [19] compared the effect of a computerized (EndoTool) versus a paper IIP (modified Portland protocol) in 300 ICU patients, showing better glucose control and nurses’ satisfaction with the EndoTool IIP. In both studies, however, it was not only the protocol that differed but also the way it was implemented (paper vs. computerized), making it difficult to assess the true value of the protocol itself.

Measurement frequency is an inherent part of an IIP and will affect glucometrics. Recently, the effect of the IIP (Yale vs. University of Washington), frequency of glucose measurements (hourly vs. every 5 min), and measurement imprecision on glycemic control efficacy was studied using a simulation model [16]. In both IIPs, the rates of hypo- and hyperglycemia and of glycemic variability increased with increasing measurement imprecision. Others investigated the performance of the IIP versus methodology of glucose measurements (blood glucose meter vs. CGM) at different levels of measurement accuracy [17]. The protocol itself proved to have a greater effect on glycemic control efficacy than the glucose measurement method, with the Yale protocol showing the best performance. However, hypoglycemia risk was lower in CGM-informed IIPs [17]. Thus, efficacy of the IIP together with performance and accuracy of the CGM device used both contribute to the success of tight glucose control. In the future, validated computerized IIPs can be guided by real-time CGM in a semi-closed loop, thereby improving efficacy, safety and reducing nursing workload.

Comparison of glucometrics between studies using different IIPs is difficult due to differences in population, target glycemia, and frequency of glucose monitoring. In addition, many different glucometrics are reported in different studies, including metrics of central tendency (mean or median glycemia, time-averaged glucose, admission glycemia, proportion of glucose values in target), metrics of extremes (percentages or episodes of hypo- or hyperglycemia), and metrics of dispersion (SD, coefficient of variation, MAGE). The robustness of glucometrics depends largely on the number of measurements per time unit used for its calculation. Accurate assessment of time in target glycemia, or in hypo- or hyperglycemia, and of glucose variability can only be done by using validated CGM methodology.

Our study has some limitations and strengths. This is a pooled data analysis of two prospective RCTs conducted at the medical ICUs, using relatively old data. Indeed, patients were recruited between 04/2004 and 03/2005 for the first study and between 07/2007 and 09/2009 for the second study. However, pooling the CGM data is justifiable in our opinion because the same CGM sensor was used in both studies and the study population and standards of care in both services were comparable. However, our results might not be applicable to a mixed or surgical ICU setting. Despite more than 63,000 CGM glucose measurements being available for analysis, due to the small number of patients and heterogeneity of groups, statistical superiority of the Yale protocol could not be proven.

A major strength, in our opinion, when comparing IIPs, is the use of CGM data which provides a complete picture of glucometrics. We did not make use of study nurses particularly focused on glucose control, but implemented our study in a routine clinical setting, allowing a more clinically relevant picture.

In summary, the use of a safe and efficient IIP is a prerequisite to correctly implement strict glycemic targets. Both IIPs have proven to balance efficacy with safety (avoid hypoglycemia and glycemic variability) and attainability (nursing workload). Overall, the modified Yale protocol provided better glucose control with more time spent in normoglycemia, less time spent in hypoglycemia, and less glycemic variability as compared to the Leuven protocol.

## Additional file

**Additional file 1: Table S1.** Baseline characteristics and glucometrics of patients treated by the Leuven versus Yale protocol, analysed according to diabetes status.

## Abbreviations

BMI: body mass index
CGM: continuous glucose monitoring
CONGA: continuous overlapping net glycemic
HBGI: high blood glucose index
ICU: intensive care unit
IIP: insulin infusion protocol
IV: intravenous
LBGI: low blood glucose index
MAGE: mean amplitude of glucose excursions
MICU: medical intensive care unit
MODD: mean of daily differences
RCT: randomized controlled trial(s)
SD: standard deviation
UZA: Universitair Ziekenhuis Antwerpen (Antwerp University Hospital)
