# Supplementary material for: A comparison of two insulin infusion protocols in the medical intensive care unit by continuous glucose monitoring
Source: Ann Intensive Care. 2016 Nov 22;6:115. doi: 10.1186/s13613-016-0214-9 (PMC5120161; doi:10.1186/s13613-016-0214-9)
Supplement: Supplementary file 1 — Additional file 1: Table S1. Baseline characteristics and glucometrics of patients treated by the Leuven versus Yale protocol, analysed according to diabetes status. [file 13613_2016_214_MOESM1_ESM.doc]

Supplemental Table: Baseline characteristics and glucometrics of patients treated by the Leuven vs Yale protocol, analysed according to diabetes status.

|  | |  | |  | **non-DM** | |  | **statistics** | **DM** |  | **statistics** |
| --- | --- | --- | --- | --- | --- | --- | --- | --- | --- | --- | --- |
|  | |  | |  | **Leuven** | | **Yale** |  | **Leuven** | **Yale** |  |
| number of patients | | | | | 9 | | 27 |  | 13 | 8 |  |
| **patient demographics** | | | | |  | |  |  |  |  |  |
|  | | men/women | | | 5/4 | | 13/14 | NS | 8/5 | 4/4 | NS |
|  | | age (years) | | | 62 ± 13 | | 67 ± 10 | NS | 60 ± 14 | 65 ± 9 | NS |
|  | | BMI (kg/m^2^) | | | 24.0 ± 5.9 | | 26.1 ± 3.4 | NS | 29.9 ± 7.1 | 27.4 ± 5.8 | NS |
|  | |  | |  |  | |  |  |  |  |  |
| **severity of illness** | | | | |  | |  |  |  |  |  |
|  | | APACHE II score | | | 28 ± 4 | | 29 ± 7 | NS | 26 ± 8 | 26 ± 4 | NS |
|  | | SOFA score | | | 10±4 | | 11±3 | NS | 10±3 | 9±3 | NS |
|  | |  | |  |  | |  |  |  |  |  |
| **insulin dose** | | | | |  | |  |  |  |  |  |
|  | | day 1 (units) | | | 59 (41-175) | | 47 (24-81) | NS | 165 (93-232) | 56 (46-96) | 0.044 |
|  | | day 2 (units) | | | 73 (25-147) | 55 (25-78) | | NS | 129 (72-187) | 71 (31-115) | NS |
|  | |  | |  |  | |  |  |  |  |  |
| **glucose parameters** | | | | |  | |  |  |  |  |  |
|  | HbA1c (%) | | | | 5.8 (5.6-6.4) | | 5.9 (5.6-6.3) | NS | 6.9 (6.2-7.3) | 6.8 (6.1-7.3) | NS |
|  | HbA1c (mmol/mol) | | | | 40 (36-46) | | 41 (38-45) | NS | 52 (44-56) | 51 (43-56) | NS |
|  | median glycemia (mg/dl) | | | | 115 (101-140) | | 117 (108-127) | NS | 136 (111-169) | 120 (108-140) | NS |
|  | % of time at glycemia | | | |  | |  |  |  |  |  |
|  |  | | < 60 mg/dl | | 1.3 (0.0-6.2) | | 0.0 (0.0-0.2) | 0.013 | 4.5 (2.3-10.7) | 2.0 (0.0-8.3) | NS |
|  |  | | 80-110 mg/dl | | 25 ± 10 | | 40 ± 15 | 0.009 | 23 ± 12 | 27 ± 10 | NS |
|  |  | | > 150 mg/dl | | 21 ± 17 | | 13 ± 8 | 0.075 (NS) | 35 ± 25 | 26 ± 21 | 0.003 |
|  |  | | > 200 mg/dl | | 7 ± 8 | | 2 ± 3 | 0.006 | 18 ± 23 | 9 ± 12 | NS |
|  |  | | 60-150 mg/dl | | 75 ± 16 | | 86 ± 8 | 0.013 | 59 ± 24 | 70 ± 20 | NS |
|  |  | | 70-180 mg/dl | | 78 ± 14 | | 94 ± 6 | <0.0001 | 63 ± 17 | 80 ± 14 | 0.054 (NS) |
|  | nr of art blood glc /day | | | | 10 ± 2 | | 9 ± 4 | NS | 10 ± 2 | 11 ± 5 | NS |
|  | glucose variability parameters | | | |  | |  |  |  |  |  |
|  |  | | standard deviation (mg/dl) | | 35 (26-54) | | 26 (21-34) | 0.076 (NS) | 63 (41-90) | 47 (34-62) | NS |
|  |  | | coefficient of variation (%) | | 32 (22-45) | | 22 (18-27) | 0.057 (NS) | 45 (29-59) | 34 (29-51) | NS |
|  |  | | IQR | | 54 (41-66) | | 34 (27-41) | 0.005 | 77 (51-124) | 53 (37-58) | 0.075 (NS) |
|  |  | | MAGE (mg/dl) | | 56 (40-116) | | 48 (35-55) | NS | 79 (49-132) | 89 (65-101) | NS |
|  |  | | MODD (mg/dl) | | 43 (30-67) | | 32 (24-38) | 0.021 | 80 (40-130) | 50 (30-59) | NS |
|  |  | | M-100 | | 6 (3-14) | | 3 (1-4) | 0.017 | 14 (7-42) | 10 (4-17) | NS |
|  |  | | CONGA1 (mg/dl) | | 17 (13-23) | | 15 (12-20) | NS | 35 (16-51) | 26 (21-36) | NS |
|  |  | | CONGA2 (mg/dl) | | 25 (17-38) | | 22 (17-30) | NS | 45 (26-75) | 37 (25-49) | NS |
|  |  | | CONGA4 (mg/dl) | | 42 (21-60) | | 29 (20-38) | NS | 64 (37-91) | 59 (36-63) | NS |
|  |  | | LBGI | | 1.9 (0.2-3.4) | | 0.7 (0.3-1.2) | NS | 3.0 (1.3-3.8) | 1.1 (0.6-2.8) | NS |
|  |  | | HBGI | | 2.1 (0.4-4.4) | | 1.2 (0.4-1.6) | NS | 4.2 (1.4-11.4) | 2.4 (1.4-3.8) | NS |
|  |  | | ADRR | | 18 (11-26) | | 8 (5-12) | 0.008 | 21 (16-48) | 27 (14-38) | NS |
|  |  | | glucose variability | | 39 (23-51) | | 27 (19-30) | 0.043 | 47 (36-82) | 37 (29-51) | NS |
